# Editorial: Molecular nutrition as preventive tool in non-communicable diseases: Mechanistic insights and risk biomarkers

**DOI:** 10.3389/fnut.2022.1005397

**Published:** 2022-09-29

**Authors:** Domenico Sergi, Laura Bordoni

**Affiliations:** ^1^Department of Translational Medicine, University of Ferrara, Ferrara, Italy; ^2^Unit of Molecular Biology and Nutrigenomics, School of Pharmacy, University of Camerino, Camerino, Italy

**Keywords:** nutrigenomics, molecular nutrition, microbiota, biomarkers, non-communicable disease (NCD), lipids, metabolism

Nutrition has a crucial role in modulating aging trajectories. Diet is not a mere source of energy, but it also affects immune functions, inflammatory status, epigenetic regulations, and gene expression (1). Also, diet affect the gut microbiome composition, thus modulating the metabolites produced by the microorganisms, which can have both positive and negative effects on health (2).

Due to their action at the level of several biochemical and molecular pathways, dietary habits have a major role in the development and progression of complex non-communicable diseases (NCD) (e.g., obesity, cardio-metabolic disorders, neurodegeneration), which represent a major burden for the modern society (3). A plethora of bioactive compounds, nutrients and dietary patterns have been described as potential discriminants of the health status (4). Some metabolites have been also proposed as risk biomarkers for non-communicable diseases (e.g., trimethylamine n-oxide) (5), thus opening interesting possibilities for prevention interventions and population risk stratification. Nevertheless, elucidating the exact molecular mechanism underpinning the effects of each dietary factor remains an ambitious goal of modern nutrition. This special issue aims at gathering new findings that might contribute to fill the gaps in this still marginally explored area.

Among relevant aspects that can be modulated by nutrition, there is hypercholesterolemia. Hypercholesterolemia, particularly when marked by an increase in small-dense LDL, represents a pivotal cardiovascular disease (CVD) risk factor. This is a direct consequence of unhealthy dietary patterns particularly rich in sugar, long-chain saturated fatty acids and an imbalance between omega-6/omega-3 ratio (6). Gora et al. investigated the hypocholesterolemic effects of two β-glucans, differing for their branching patterns, in a zebrafish model of diet-induced hypercholesterolemia. The high-cholesterol diet not only induced an increase in circulating LDL cholesterol, but also elicited changes in the fish intestine at the transcriptional level, indicative of suppressed cholesterol biosynthesis, endoplasmic reticulum stress and mitochondrial dysfunction. On the contrary, the inclusion of β-glucans derived from either microalga *Phaeodactylum tricornutum* or oat (PromOat^®^) countered the increase in LDL-cholesterol. Additionally, β-glucans restored the expression of several genes altered by dietary cholesterol, including those linked with endoplasmic reticulum stress. Thus, this study provides novel mechanistic insights on the effects of β-glucans cholesterol homeostasis *via* the modulation of the intestinal transcriptome. Remarkably, the hypocholesterolemic effects exerted by β-glucans were comparable to those elicited by simvastatin, a cholesterol-lowering drug, confirming the potential of these dietary fibers as an attractive therapeutic tool to dampen the aberrations in lipid metabolism undelaying an increase in cardiovascular risk.

Obesity represents a risk factor for a plethora of diseases, including type 2 diabetes and CVD (7), which are also associated with telomere shortening (8), suggesting and interplay between obesity and telomere length. In a systematic review and meta-analysis, Khosravaniardakani et al. reported that obesity negatively affects telomeres causing their shortening in leukocytes. The potential drivers of the effects of obesity on telomere length may rely oxidative stress, inflammation and hyperleptinemia, all hallmarks of excess body weight and impaired cardiometabolic health. Even though the cause-effect relationship between obesity and telomere shortening remains to be fully elucidated, telomere length may represent a potential biomarker to monitor the impact of obesity on aging.

The gut microbiota composition and its metabolic activity are key discriminants in shaping health trajectories and have been associated with the pathogenesis of obesity and CVD (9). Additionally, the gut microbiota functionality and composition is strongly influenced by diet (10). In this respect, Lakshmanan et al. investigated the impact of dietary factors and gut microbiota composition of CVD development in obese Qatari adults as part of an observational study. Authors reported that individuals at CVD risk consumed less vitamin D, which was associated with CVD risk itself. Unexpectedly, study participants with higher CVD risk had a lower intake of trans-fat and saturated fat. Despite no differences in the gut microbiota diversity, individuals with a no CVD risk displayed an increased relative abundance of Ruminococcus, compared to study participants at CVD risk. Within the genus Ruminococcus authors identified *R. callidus* and an unclassified species from the Ruminococcaceae family as the key contributors to the increase in Ruminococcus in obese individuals with no CVD risk. The gut microbiota metabolic pathways related to taurine, hypotaurine, and lipoic acid metabolism were upregulated in the CVD relative to the no CVD risk group. Finally, the relative abundance of Ruminococcus positively correlated with the intake of monounsaturated fat, vitamin D, vitamin A and proteins. This pilot study suggests the genus Ruminococcus as a novel biomarker of CVD risk, but further confirmations are warranted.

Aging can also affect brain functions, as proven by the shift of the microglia, the immune cells of the brain, toward a pro-inflammatory phenotype. Microglia activation has been linked with neurodegenerative diseases (11) as well as obesity-associated hypothalamic dysfunction (12). Dietary fibers can affect the gut-brain axis *via* the products of its fermentation, like short-chain fatty acids (SCFAs), that may influence microglia activation and neuroinflammation. Vailati-Riboni et al. investigated the impact of inulin on microglial activation in aged mice. Single-cell RNA sequencing and gene expression analysis showed an increased proportion of activated microglia with a pro-inflammatory phenotype in aged mice in parallel with a decrease in the levels of SCFAs in cecum of these animals. The supplementation of aged mice with dietary inulin, along with an increase in SCFAs in cecum, counteracted the pro-inflammatory activation status of microglia. This effect was marked by a restoration of microglia gene expression profile and TNFα secretory pattern to resemble that of adult mice. Authors also reported sex differences, with aged females having lower levels of SCFAs in cecum relative to males. Thus, the supplementation with fermentable dietary fiber may represent a potential strategy to offset or mitigate age-related shift in microglia toward a pro-inflammatory phenotype, a protective effect that may depend of the metabolites derived from fiber formation and the modulation of the gut-brain axis.

In conclusion, new hints on pathways activated by nutrition as well as biomarkers able to trace the impact of diet on health are emerging, corroborating a potential role of nutrigenomics in NCD prevention. Future prospectives in this research field include elucidating the complex role of the microbiome in mediating the impact of nutrition on health parameters; understanding how both the microbiome and genetic determinants define individual responses to nutritional interventions; the identification of molecular biomarkers able to trace the footprint of the environmental exposome (including diet) and monitor its impact on human health. Ultimately, the development of tools providing a deeper molecular phenotyping of an individual can help to dissect the complexity of the relationship between nutrition and health. This, in turn, will pave the way for the development of personalized nutritional interventions and tailored prevention strategies, effective and devoid of side effects.

## Author contributions

LB and DS: conceptualization, drafting, and revision of the manuscript. All authors contributed to the article and approved the submitted version.

## Conflict of interest

The authors declare that the research was conducted in the absence of any commercial or financial relationships that could be construed as a potential conflict of interest.

## Publisher's note

All claims expressed in this article are solely those of the authors and do not necessarily represent those of their affiliated organizations, or those of the publisher, the editors and the reviewers. Any product that may be evaluated in this article, or claim that may be made by its manufacturer, is not guaranteed or endorsed by the publisher.
